# Structures, stabilities, and electronic properties of defects in monolayer black phosphorus

**DOI:** 10.1038/srep10848

**Published:** 2015-06-02

**Authors:** Xi-Bo Li, Pan Guo, Teng-Fei Cao, Hao Liu, Woon-Ming Lau, Li-Min Liu

**Affiliations:** 1 Beijing Computational Science Research Center, Beijing 100084, China; 2 Chengdu Green Energy and Green Manufacturing Technology R&D Center, Chengdu, Sichuan, 610207, China

## Abstract

The structures, stabilities, and electronic properties of monolayer black phosphorus (M-BP) with different kinds of defects are investigated within the frame of density-functional theory. All the possible configurations of defects in M-BP are explored, and the calculated results suggest that the stabilities of the configurations with different kinds of defects are greatly related to broken bonds, structural deformation and the character of the bonding. The configurations with two or three vacancies are energetically more favorable than the ones with a single vacancy. Meanwhile, the doping of two foreign atoms, such as sulfur, silicon or aluminum, is more stable than that of the corresponding single dopant. The electronic properties of M-BP are greatly affected by the types of defects. The single S-doped M-BP not only retains the character of a direct semiconductor, but it also can enlarge the band gap by 0.24 eV relative to the perfect one. Such results reveal that the defects not only greatly affect the electronic properties, but they also can be used as an effective way to modulate the band gap for the different applications of M-BP in electronic devices.

Two-dimensional materials, due to their novel electronic and optical properties, have attracted numerous studies for decades. As one of these materials, although graphene exhibits plenty of advantages, and it has dominated the two-dimension research field for many years^1^,^2^,^3^,^4^, the gapless nature of graphene greatly limits its applications in electronic devices. Thus many groups have made efforts to explore other single layer materials, such as boron nitride^5^, or transition metal dichalcogenides (TMDs)^6^,^7^,^8^,^9^.

One of the widely studied semiconductor materials is black phosphorus (BP)^10^,^11^,^12^,^13^,^14^,^15^,^16^,^17^, which possesses puckered layered structure of one elemental phosphorus allotrope. In bulk BP^18^,^19^, different layers interact through the weak van der Waals (vdW) force, a configuration which enables one to exfoliate a single layer from the bulk material. Recently, monolayer black phosphorus (M-BP), namely phosphorene, has been fabricated by several research groups^20^,^21^. Further theoretical calculations show that the band gap of BP is layer dependent with 0.30 eV in bulk BP and 0.90 eV in monolayer BP^21^,^22^, which is relatively smaller than the corresponding values of 0.35 eV and 2.00 eV^23^ in experiments, respectively. Its tunable property of band gap and the super electronic performance largely enhance its promising applications in many electronic devices, such as lithium-ion batteries^24^,^25^,^26^, thin-film solar cells^27^,^28^, channel material in field-effect transistors and thermoelectric devices^29^,^30^.

Because of its superior electronic performance and structure-dependent novel phenomenon, BP has immediately attracted great attention and becomes one of the most research hotspots. Guo *et al.*^31^ investigated nanotubes of BP and predicted that phosphorene was a promising candidate for future nanoelectronic devices. Several groups^32^,^33^ studied the strain effect on the band gap of BP, and they revealed that BP possessed a rich variety of electronic and structural transformations under different pressures. Gong *et al.*^34^ systematically investigated the possible metal contacts to monolayer BP using DFT method and proposed several future device applications of BP. Zhu *et al.*^35^ did a detailed research on semiconducting layered blue phosphorus, and they indicated that it would be as useful as BP in functional materials. Liu *et al.*^36^ explored the effect of grain boundary and nanoribbons on the electronic structures, and showed that there were no deep gap states in GB and the deep gap states in the nanoribbons could be eliminated by passivation. Dai *et al.*^27^ reported the effect of stacking order on BP’s band gap and predicted its potential applications in thin-film solar cell. Despite all the advances in research on BP, one area that requires further studies is defects formed on the material during the fabrication process. As the defects can’t be avoided in the two dimensional BP fabrication process as in other two dimensional materials^37^,^38^, the electronic property and carrier mobility of M-BP may be affected by the vacancies created by these defects. Meanwhile, foreign atom doping is one of the accessible ways to tailor the electronic properties of M-BP. Thus, it is greatly necessary to examine how the defects affect the structural and electronic properties of M-BP.

In this paper, we carry out the first-principles calculations to explore the intrinsic relationship between the structures, stabilities, and electronic properties of defects (vacancy, sulfur doping, silicon doping, or aluminum doping) in M-BP. Through the examination of formation energies, the most stable configuration with each kind of defect at different defect degrees is determined. The study reveals a number of interesting observations. Firstly, the broken bonds, structural deformation or the character of bonding make divacancy or two dopants M-BP even more stable than the corresponding single one. Secondly, the electronic properties can be modulated by different kinds of defects and their densities. These results provide detailed information for future applications of M-BP.

## Computational details

The calculations were carried out within the frame of density functional theory (DFT), as implemented in the Vienna ab-initio simulation package (VASP)^39^. The exchange–correlation potential was described by the generalized gradient approximation (GGA) functional of Perdew, Burke, and Ernzerhof (PBE)^40^. The van der Waals (vdW) correction proposed by Grimme (DFT-D2)^41^ was included in our calculations. The plane wave cut-off energy of 400 eV was chosen. A 3 × 3 × 1 k-point sampling based on Gama-centered Monkhorst-Pack scheme^42^ was used for the all structural relaxation. The structural optimization process was finished until the force exerted on each atom was less than 10^−2^ eV/Å and the total energy change was less than 10^−5^ eV. Meanwhile, a vacuum space of 15 Å was added in the unit cell to avoid the interaction between the repeated unit cells. A 4 × 4 × 1 and a 3 × 3 × 1 supercell M-BP was adopted to accommodate vacancies and foreign atoms doping, respectively.

To explore the relative stability of the configurations, we define the formation energies, E_*fv*_, of the vacancies M-BP as the following equation,


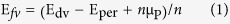


E_dv_ is the total energy of M-BP with vacancies, and E_per_ represents the total energy of perfect M-BP. *n* stands for the number of the removed phosphorus atoms and μ_P_ is the chemical potential of bulk phosphorus. In order to know the relative stabilities of foreign atom substitution, the formation energies of S-doped, Si-doped or Al-doped M-BP are calculated by the following equation:


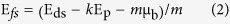


Where E_ds_ is the total energy of M-BP doped with the number *m* S, Si or Al atoms. E_p_ is the energy of a single P atom in the perfect M-BP. *K* and *m* stand for the number of P, doped S, Si or Al atoms in the doped M-BP configuration, respectively. μ_b_ is the chemical potential of bulk sulfur, silicon or aluminum.

## Results and discussions

### Properties of Perfect M-BP

**Figure 1:**
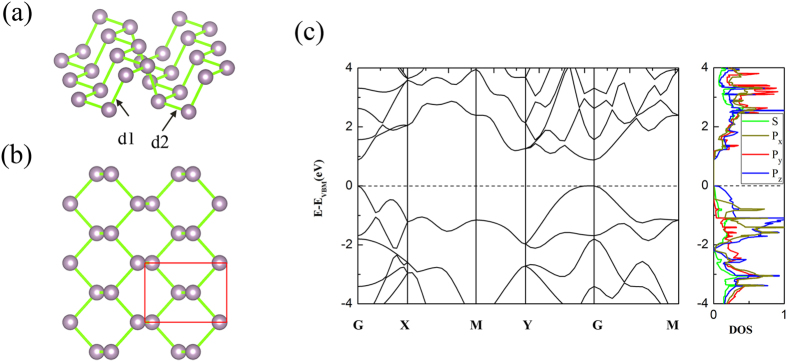
The atomic structure and electronic properties of perfect M-BP : (**a**) Side view and (**b**) top view of atomic structure. The red rectangle in (**b**) denotes the primitive cell of M-BP. (**c**) band structures and partial DOS. The Fermi level is set to zero.

As shown in Fig. 1, the M-BP is composed of buckled hexagons, along with a rectangular symmetry. The P atoms are subdivided into two sublayers, forming an armchair-like pattern. There are three bonds for each P atom in M-BP: two bonds are formed by the P atoms in the same layer with a distance of d_1 _= 2.25 Å, and the third bond is between the interlayer P atoms with a distance of d_2_ = 2.22 Å. There are two types of angles between the nearest neighbor P atoms: two angles are 103.75° between P atoms in different layers and the other one is 96.28° between P atoms in same layer. As shown in Fig. 1, the M-BP possesses a direct band gap of 0.88 eV, which is about 0.58 eV larger than the bulk, as other PBE results reported in the previous work^19^,^43^. The calculated band gap of M-BP is smaller than the experimental value of 2.0 eV^23^, which should be the reason that pure DFT usually underestimates the band gap of semiconductor because of self-interaction error. Both the conduction band minimum (CBM) and the valence band maximum (VBM) are located at the G point for M-BP, a situation which is very beneficial for the electron transport. Charge distributions of VBM are mainly located at the *p*_*z*_ orbital of the P atoms in different planes, and those of CBM are mainly located at the *p* and *s* orbitals of the P atoms in the same plane^36^, another observation indicating the excellent transport properties.

### Atomic structure of defects in M-BP

**Figure 2:**
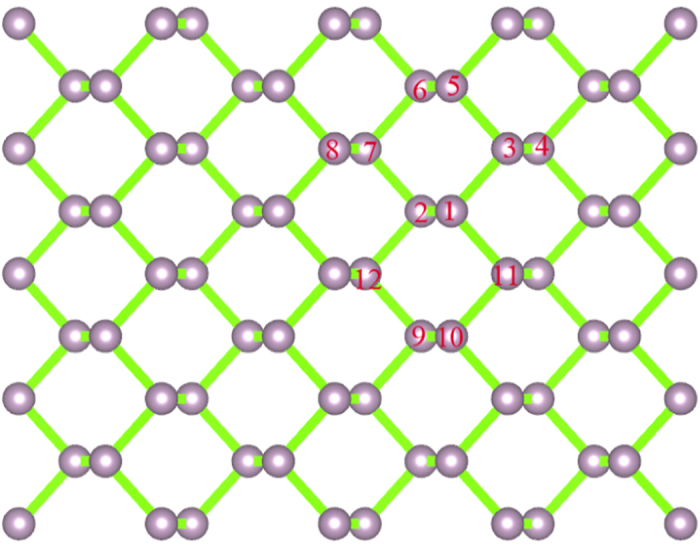
Schematic description of the vacancy and the foreign atom doped M-BP . Here, violent balls represent the P atoms of M-BP. The configuration of single vacancy M-BP can be obtained by removing P_1_ atom. The various configurations of the divacancy can be obtained by removing P_1_-P_2_, P_1_-P_3_, P_1_-P_4_, P_1_-P_5_, P_1_-P_6_, P_1_-P_7_, and P_1_-P_8_ atoms, respectively. The corresponding doped configurations are obtained by replacing the P atoms by the doping atoms.

To explore the characteristics of the vacancy defects or foreign atom (sulfur, silicon or aluminum) doping systems of M-BP, we consider the possible configurations. Due to the equivalency of all the phosphorus atoms, there is only one kind of single vacancy or foreign atom doping M-BP configuration. As shown in Fig. 2, the configuration with only the P_1_ atom removed is similar to the one with only P_2_ removed. When the number of vacancies or doping atoms becomes two, there are several possible configurations, such as P_1_-P_2_, P_1_-P_3_, P_1_-P_4_, P_1_-P_5_, P_1_-P_6_, P_1_-P_7_ and P_1_-P_8_. The typical defect of P_1_-P_2_ vacancy (foreign atom doped) M-BP is shown in Fig. 2. Because of the equivalent of the P_1_-P_4_ and P_1_-P_7_, only P_1_-P_4_ defect M-BP is considered in the following. And for three vacancies, only four kinds of configurations are considered, e.g., P_1_-P_3_-P_11_, P_1_-P_2_-P_7_, P_1_-P_6_-P_9_, P_1_-P_5_-P_10_ vacancies M-BP. For simplicity, the M-BP with P_n_-P_m_ vacancy is labeled as V_n-m_, and the M-BP with sulfur (silicon, aluminum) atoms doping is labeled as S_n-m_ (Si_n-m_, Al_n-m_). Here, n and m represent the index of P atoms, which are removed from the perfect one or substituted with the other atoms. For example, the M-BP with the type of P_1_-P_2_ vacancy is labeled as V_1−2_ M-BP.

### The stabilities and electronic structures of M-BP with different vacancies

**Figure 3:**
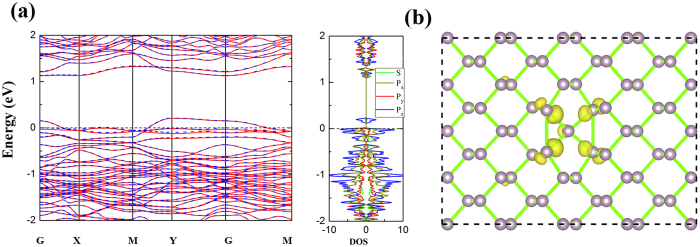
The electronic properties of V_1_ M-BP : (**a**) Band structures and partial DOS and (**b**) spin density distribution of V_1_ M-BP. The isovalue is set to 0.0005 e/bohr^3^. The Fermi level is set to zero. In (**a**), the solid red line and the dashed blue line indicate the spin up and down, respectively.

For single vacancy in M-BP (V_1_), an obvious structural deformation occurs. The P atom (P_2_ atom) below the vacancy site (removed P_1_ atom) moves toward the direction of the vacancy site by a distance of 0.37 Å, forming two bonds with nearby P atoms in the layer of vacancy with a distance of 2.47 Å. The other two with nearby P atoms are in another layer of P_2_ atom with a distance of 2.33 Å. The formation energy of V_1_ is 2.00 eV. The ground state of V_1_ in M-BP is spin-polarized with a magnetic moment of 0.21 μ_B_. As shown in Fig. 3(a), the calculated band structures indicates that the valance band of V_1_ M-BP is split into two bands near the X, M and G points, and also there is one band across the Fermi level. As shown in Fig 3(a,b), partial DOS shows that the spin polarization is located at the *p*_*z*_ orbital, which mainly distributes around the P atoms near the vacancy.

**Figure 4:**
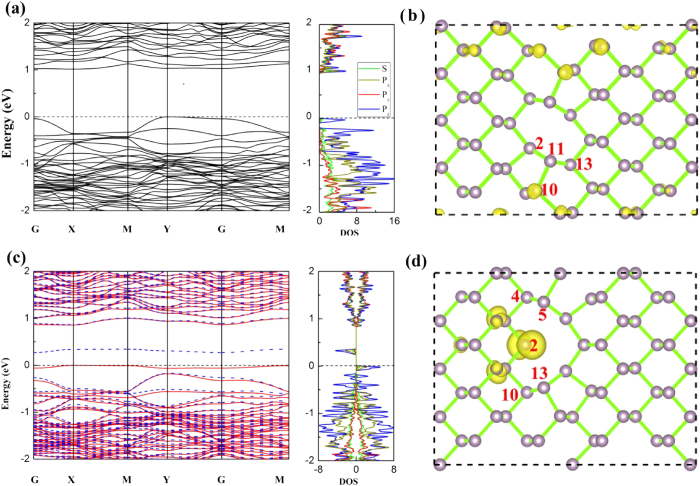
The electronic structure of V_1-3_ M-BP and V_1−3−11_ M-BP. Band structures and partial DOS are shown in (**a**) and (**c**), and electron distributions of highest occupied molecular orbital (HOMO) are shown in (**b**) and (**d**), respectively. The isovalue of the electron density is set to 0.0025 e/bohr^3^. The upper panel corresponds to the V_1−3_, and the lower panel represents V_1−3−11_ M-BP. The Fermi level is set to zero. The solid red line and the dashed blue of band structures indicate the spin up and down, respectively.

When there are two or three vacancies in M-BP, several typical configurations exist. The several typical configurations of divacancy (also two dopants in next section) are shown in Fig. 2. The relative stabilities of these configurations are examined, and the corresponding formation energies are shown in Table 1. Obviously, all divacancy configurations (except V_1−8_) are more stable than single-vacancy configurations. Furthermore, the V_1-3_ vacancies M-BP is the most stable one among all of them, with the formation energy of 0.72 eV/vacancy. The high stability of V_1−3_ is greatly correlated with the large structural deformation. When two P atoms (P_1_ and P_3_ atoms) are removed from the perfect M-BP, the P_11_ atom moves towards the P_2_ atom, and the distances between the P_11_ and P_2_, P_11_ and P_10_, P_11_ and the P_13_ atom below P_11_ atom become 2.39 Å, 2.27 Å, 2.25 Å, respectively (see Fig. 4(b)).

Those deformations bond every P atom to three nearby P atoms, forming new sp^3^ hybridization by structural deformation, and this should also be one reason that V_1−3_ vacancies M-BP is more energetic favorable than the single vacancy case. The newly formed sp^3^ hybridization for P atom leads to the delocalization of the valance band state. The impurity state mainly originates from the *p*_*z*_ orbital of the P atoms, which is far from the vacancies, as shown in Fig. 4(a,b). In addition, there is also another unoccupied impurity state below the CBM mainly distributing around the defect sites. As a result of the V_1−3_ vacancies, the band gap of the defect M-BP becomes an indirect one from Y point to G point with a value of 1.02 eV.

Among all three vacancies in M-BP, the calculated results show that the configuration of V_1−3−11_ M-BP, with the three vacancies in the same armchair line, is the most stable configuration. It should be noted that P_3_ and P_11_ are the equivalent P atoms nearest to the P_1_ atom, and configuration V_1−3_ is the most stable configuration of divacancy M-BP. The formation energy of V_1−3−11_ vacancies is 0.68 eV, which is smaller than that of the two vacancies configuration V_1_−_3_. Here, the stability of M-BP with different number of vacancies is discussed. We divide the formation of the vacancies into two parts: the P atoms are removed from perfect M-BP, and the M-BP with vacancies is transformed to a stable state. The corresponding energy changes are broken bond energy and deformation energy, respectively. Once the V_1_ ,V_1−3_, V_1−3−11_ configurations are formed, the number of broken bonds are 3, 5/2, 7/3 per vacancy and the corresponding broken energy are 2.36 eV, 1.51 eV, 1.29 eV per vacancy, respectively, showing strong positive correlation between broken bond energy and the number of broken bonds per vacancy. In addition, the deformational energies of V_1_, V_1−3_, V_1−3−11_ configurations are −0.36 eV, −0.79 eV, −0.61 eV per vacancy, respectively. Thus, the common contributions of the two parts determine the stabilities of the most stable configuration with different density of vacancy.

Different from one and two vacancies case, V_1−3−11_ M-BP exhibits more occupied spin up states than those that spin down below the Fermi level (see Fig. 4(c)), leading to a net spin magnetic moment of 1 μ_B_. And the magnetic moment mainly comes from the *p*_*z*_ orbital of P atoms, which includes the P_2_ atom and P atoms around it (see Fig. 2 and Fig. 4(d)). This distribution of spin density of V_1−3−11_ is also greatly related with the structural deformation of V_1−3−11_ M-BP. The P_4_ atom (P_13_ atom below P_11_ vacancy site) moves toward the P_5_ (P_10_) atom to keep the sp^3^ hybridization. However, the P_2_ atom below the P_1_ vacancy only bonds to the two nearest P atoms, which creates a dangling bond in the P_2_ atom. In addition, there is also one unoccupied localized state below the CBM, and the V_1−3−11_ M-BP owns an even larger indirect band gap of 1.20 eV (from Y point to G point).

In all, the stabilities and electronic properties of M-BP with different number of vacancies are investigated. The formation energies with different vacancies indicate that the two and three vacancies are more stable than the single vacancy. For example, the formation energy of V_1−3_ M-BP is 1.28 eV lower than that of V_1_ M-BP, which indicates that the second P atom (P_3_ or P_11_) can easily come out of M-BP once the first P atom (P_1_) was removed.

### The stabilities and electronic structures of sulfur, silicon or aluminum doped M-BP

**Figure 5:**
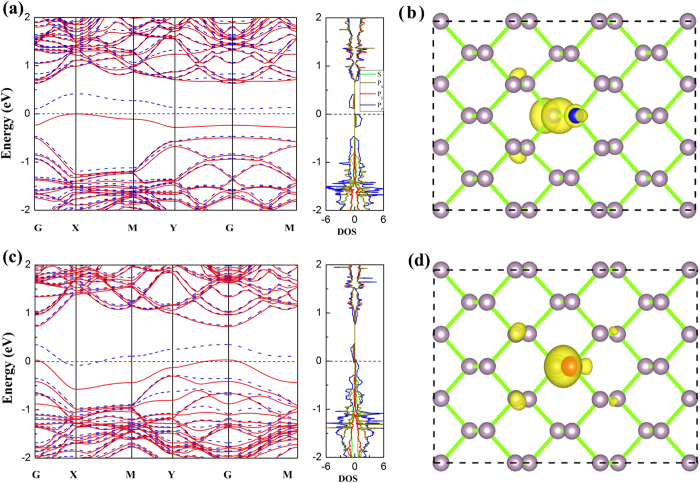
The electronic structure of single S or single Si doped M-BP M-BP : Band structures and partial DOS are shown (**a**) and (**c**), and electron distributions of HOMO are shown in (**b**) and (**d**). The upper panel corresponds to the S_1_, and the lower panel represents Si_1_ M-BP. The isovalue is set to 0.004** **e/bohr^3^. The Fermi level is set to zero. The solid red and the dashed blue lines of band structures indicate the spin up and down, respectively.

One of the most effective techniques to tune the electronic property of two-dimensional systems is to dope foreign atoms. Sulfur, aluminum and silicon atoms have one more, two less and one less electron than P atom, respectively. They are expected to induce extra states and tailor the electronic properties of M-BP. In the following, the stabilities and electronic properties of M-BP with S, Si or Al doping are discussed. The electronic structures of M-BP with one dopant, sulfur or silicon, are examined firstly. In the case of the single S-doped M-BP, the calculated band structures are shown in Fig. 5(a). The S-impurity state is in the middle of the band gap, which is split into two states: one occupied spin up, the other unoccupied spin down. Thus the ground state of single S-doped M-BP is spin-polarized with a magnetic moment of 1.0 μ_B_. Those results indicate that S is not a n-type dopant, as a good n-type dopant should introduce filled states below the CBM closely. The S-doped M-BP remains a direct semiconductor with a band gap of 1.12 eV at G point. The impurity states of the single S-doped M-BP mainly occupy the *p*_*z*_ orbital in partial DOS (see Fig. 5(a)). The magnetic moment is mainly localized around the P atom below the doped S atom (see Fig. 5(b)). The localized magnetism around the P atom and the occupied induced state suggest that the unpaired electrons are mainly located at around the P atom below the S atom. The magnetic distribution induced by the S atom should come from the different numbers of electrons between the S and P atoms. Since the S atom has six valance electrons, when replacing one P with S, the S atom bonds to the two P atoms in the same layer, which makes the *p* orbital of the S atom fully occupied. Thus the P atom below the sulfur atom is unable to form a bond with the S atom, leaving one unpaired electron. Then the magnetic moment comes from the extra valance electron of the S atom, leaving one electron of the P atom unpaired.

The electronic structure of M-BP doped with one silicon atom is also examined. Similar to the S-doped M-BP, the Si-impurity state of Si-doped M-BP is observed in the middle of the band gap and split into two states: partially occupied spin up and down, leading to a spin polarization with a magnetic moment of 0.99 μ_B_. Obviously, single S-doped and Si-doped M-BP exhibits different electronic structures. The single Si-doped M-BP show two states across the Fermi level, similar to that of V_1_ M-BP. As shown in Fig. 5(c), the partial DOS shows that the impurity states mainly occupy the *p*_*z*_ orbital, similar to that of S-doped M-BP. The magnetic moment is mainly located around the doped Si atom, as shown in Fig. 5(d). The main reason should come from the fact that silicon has one less electron than phosphorus, and the unpaired electron is mainly located around the silicon atom. In additional, Si is not an effective p-type dopant, as a good p-type dopant should introduce unfilled states above the VBM closely, but Si-doped M-BP does not own this characteristic as discussed above (see Fig. 5(c)).

**Figure 6:**
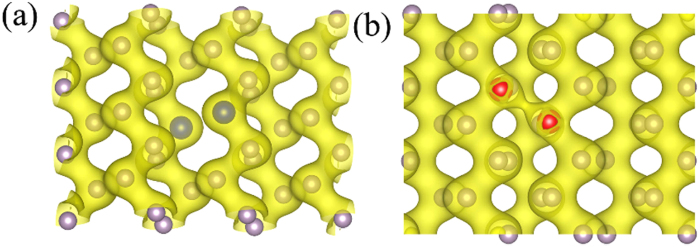
Total electron density in S_1−2_ (**a**) and Si_1−8_ (**b**) doped M-BP. The isovalue of the electron density is set to 0.06 e/bohr^3^. The P, S, Si atom are represented by grey, blue, red ball respectively.

In order to further understand how the electronic structure is affected by the doping density, M-BP with two dopant atoms is further investigated. Here, we consider the typical configurations with bi-S-doped and bi-Si-doped M-BP. Among all the typical double atom doped configurations, the most stable doping configurations for bi-S-doped and bi-Si-doped M-BP are S_1−2_ and Si_1−8_, respectively. The atomic structure of S_1−2_ M-BP is explored firstly. As shown in Fig. 6(a), the two doping S atoms are close to each other. Each single S atom needs to bond with two P atoms to form an sp^3^ hybridization, while each P atom has to bond with three P atoms to form an sp^3^ hybridization in perfect M-BP. Thus the effective utilization of the two bonds of S atom is critical for the most stable bi-S-doped M-BP. When the two S atoms are doped into the neighbor sites of the M-BP, every S atom can form two bonds with the P atom whereas there is no bonding between the S atoms. Meanwhile the distance between the S atoms in S_1−2_ M-BP is enlarged to 3.31 Å to avoid the repulsive interaction between them.

Nevertheless, the most stable configuration of bi-Si-doped M-BP is Si_1−8_ M-BP, which is greatly different from the one of bi-S doping, as shown in Fig. 6(b). The two doping atoms do not like to stay closely. The difference between these two dopants mainly comes from the distinct electronic structures. In addition, it should be noted that the deformation of the bi-Si-doped M-BP configurations Si_1−8_ is rather large. The Si atoms move toward to the P atoms below them. Meanwhile, the P atoms move in the same direction, even breaking the two-layer structure in the process. The distance between the two Si atoms is 2.63 Å, which is obviously shorter than the one between the P atoms at the corresponding sites in perfect M-BP (3.50 Å).

We further extend our study to discuss the stabilities of the S-doped and Si-doped M-BP. The calculated formation energies of different doped M-BP are shown in Table 1. The doping of the first S atom into M-BP needs extra energy of 0.09 eV. After doping of the second S atoms, the formation energy becomes negative, which suggests that the second S atom is easier to dope. Different from those of the S-doped case, the formation energies of several bi-Si-doped M-BP are always positive, thus making S atom easier to dope into M-BP than the Si atom.

**Figure 7:**
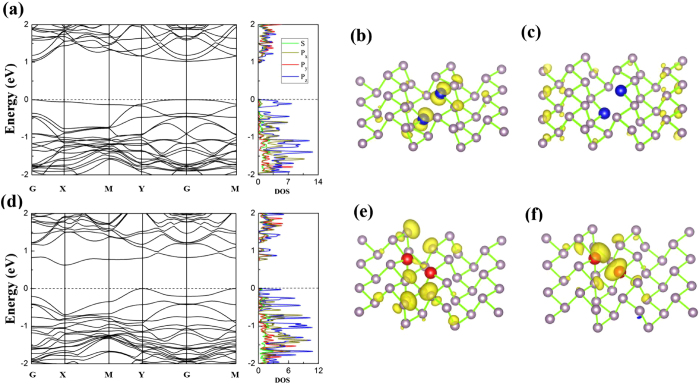
The electronic structures of S_1−2_ and Si_1−8_ M-BP : Band structure and partial DOS ((**a**) and (**d**)) and electron distributions of HOMO ((**b**) and (**e**)) and LUMO ((**c**) and (**f**)). The upper panel ((**a**)–(**c**)) corresponds to properties of S_1−2_ M-BP, and the low panel ((**d**)–(**f**)) represent the results of Si_1−8_ M-BP. The isovalue of the electron density is set and 0.004 e/bohr^3^. The Fermi level is set to zero. The P, S, and Si atoms are represented by grey, blue, and red ball respectively.

In the following, the electronic properties of the most stable configurations of bi-doped M-BP are also explored, as shown in Fig. 7. Unlike single S-doped or Si-doped configurations, S_1−2_ and Si_1−8_ M-BP do not exhibit any magnetism. As shown in Fig. 7(a), there is an impurity state at the top of the valence band. The impurity electronic state of S_1−2_ M-BP is mainly delocalized around the doped-S atom. The band structures of S_1−2_ M-BP show that the doped M-BP becomes an indirect semiconductor with a band gap of 1.18 eV, which is about 0.3 eV larger than the perfect one. We also take this as an example to explore how the supercell size affects the band gap. The band gap of S_1−2_ M-BP with 4 × 4 × 1 supercell is 1.00 eV, about 0.18 eV smaller than that with a 3 × 3 × 1 supercell. This could be understood by the impact of the density of the S_1−2_ type dopant, i.e., the smaller density of S_1−2_ type dopant and the smaller band gap. This indicates the density of dopants could impact the electronic structures of M-BP in another way.

The band gap of Si_1−8_ M-BP is an indirect one with a value of 0.99 eV. Si_1−8_ M-BP exhibits two impurity states with one occupied near the Fermi level and another unoccupied below CBM (see Fig. 7(d)). The electronic states of Si_1−8_ M-BP impurity states are shown in Fig. 7(e,f), respectively. The occupied impurity state mainly distributes at the P atoms in two lines, which contains the silicon atoms, whereas the unoccupied impurity state mainly localizes at the silicon atoms.

**Figure 8:**
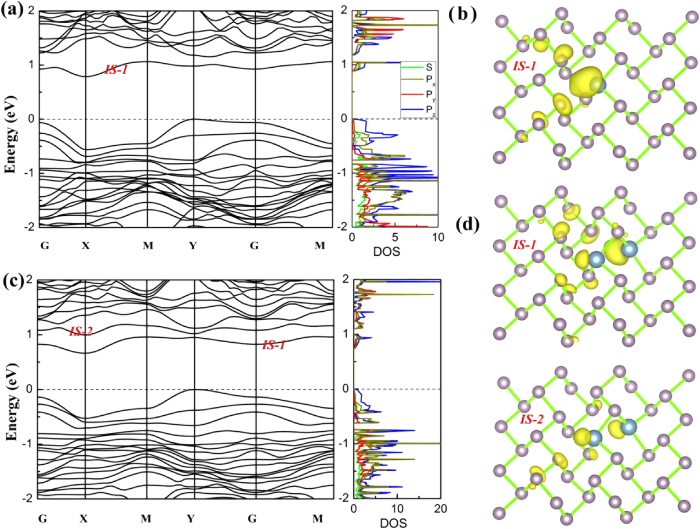
The electronic structure of Al_1_ or Al_1−4_ doped M-BP : Band structures and partial DOS are shown (**a**) and (**c**), and electron distributions of impurity states are shown in (**b**) and (**d**). The upper panel corresponds to the Al_1_, and the lower panel represents Al_1−4_ M-BP. The isovalue is set to 0.004 e/bohr^3^. The Fermi level is set to zero. The light blue balls indicate Al atoms.

Finally, the stabilities and electronic properties of M-BP with Al doping are also discussed. As listed in Table 2, the formation energies indicate that the Al_1−4_ M-BP is the most stable configuration of single-Al and bi-Al doped case. Furthermore, the formation energies indicate that the Al atoms are easier to dope into M-BP than the sulfur atoms. Moreover, the electronic properties of A_1_ and A_1−4_ M-BP are also explored, as shown in Fig. 8. As the Al atom has two less electrons than the P atom, there will be one fewer occupied state once one Al atom is doped into M-BP, as shown in Fig. 8(a,c). The unoccupied impurity state of Al_1_ M-BP is below the VBM (Fig. 8(a)) and it is mainly localized around the Al atom as shown in Fig. 8(b). The band structures of Al_1_ M-BP show that the doped M-BP still nearly remains a direct semiconductor with a band gap of 1.15 eV, locating at Y point. Al_1−4_ M-BP has a smaller band gap, about 1.12 eV. There are also two unfilled impurity states emerging below the CBM (Fig. 8(c)), mainly localizing around the two Al dopants (Fig. 8(d)).

## Conclusion

In summary, the structures, stabilities and the electronic properties of vacancy, S-doped, Si-doped, Al-doped M-BP are investigated by first-principle calculations. The most stable configurations of vacancy and doping are explored. The results reveal that the stabilities of the configurations are mainly determined by broken bonds, the structural deformation and the character of bonding. The divacancy or bi-doped configurations are more stable than single-vacancy or single-doping, and the degree of difficulty for doping atom into M-P is from Al to Si. All kinds of defects discussed could tune the electronic properties of M-BP. For single S or Si atom doping, the spin density is mainly localized around the doping atom, and there are impurity states among the band gap, occupying the *p*_*z*_ orbital and in the middle of the band gap. Our results indicate that the band gap could be modulated by introducing vacancies or dopants. Those results should be useful for the design of novel electronic devices with M-BP.

## Additional Information

**How to cite this article**: Li, X.-B. *et al.* Structures, stabilities, and electronic properties of defects in monolayer black phosphorus. *Sci. Rep.* **5**, 10848; doi: 10.1038/srep10848 (2015).

## Figures and Tables

**Table 1:** The calculated formation energies of several different vacancies and sulfur, silicon or aluminum doped M-BP, based on Eq. 1 and Eq. 2.

	**V_1_**	**V_1−2_**	**V_1−3_**	**V_1−4_**	**V_1−5_**	**V_1−6_**	**V_1−8_**
**Vacancies (eV)**	2.00	1.60	0.72	1.04	1.48	0.73	2.07
	**V**_**1**−**3**−**11**_	**V**_**1**−**2**−**7**_	**V**_**1**−**6**−**9**_	**V**_**1**−**5**−**10**_			
	0.68	1.08	1.02	1.01			
**S-doped (eV)**	**S**_**1**_	**S**_**1**−**2**_	**S**_**1**−**3**_	**S**_**1**−**4**_	**S**_**1**−**5**_	**S**_**1−6**_	**S**_**1−8**_
	0.09	−0.70	−0.64	−0.07	−0.49	−0.05	−0.18
**Si-doped (eV)**	**Si**_**1**_	**Si**_**1−2**_	**Si**_**1−3**_	**Si**_**1−4**_	**Si**_**1−5**_	**Si**_**1−6**_	**Si**_**1−8**_
	0.70	0.64	0.87	0.69	0.44	0.71	0.23
**Al-doped (eV)**	**Al**_**1**_	**Al**_**1−2**_	**Al**_**1−3**_	**Al**_**1−4**_	**Al**_**1−5**_	**Al**_**1−6**_	**Al**_**1−8**_
	−1.10	−0.58	−0.44	−1.23	−1.17	−1.21	−0.96

**Table 2:** The number of broken bonds, bond broken energies, deformation energies, and formation energies of most stable vacancies M-BP per vacancy.

**Vacancies (eV)**	**N**	**E_*bb*_**	**E_*df*_**	**E_*f*_**
**V**_**1**_	3/1	2.36	−0.36	2.00
**V**_**1**−**3**_	5/2	1.51	−0.79	0.72
**V**_**1**−**3**−**11**_	7/3	1.29	−0.61	0.68

## References

[b1] Novoselov K. S. *et al.* Electric Field Effect in Atomically Thin Carbon Films. Science 306, 666–669, doi:10.1126/science.1102896 (2004). 15499015

[b2] Geim A. K. & Novoselov K. S. The rise of graphene. Nat. Mater 6, 183–191, doi:10.1038/nmat1849 (2007). 17330084

[b3] Rasuli R., zad A. I. & Ahadian M. M. Mechanical properties of graphene cantilever from atomic force microscopy and density functional theory. Nanotechnology 21, 185503, doi:10.1088/0957-4484/21/18/185503 (2010). 20388969

[b4] Balandin A. A. Thermal properties of graphene and nanostructured carbon materials. Nat. Mater 10, 569–581, doi:10.1038/nmat3064 (2011). 21778997

[b5] Dean C. R. *et al.* Boron nitride substrates for high-quality graphene electronics. Nat. Nano 5, 722–726, doi:10.1038/nnano.2010.172 (2010). 20729834

[b6] Chhowalla M. *et al.* The chemistry of two-dimensional layered transition metal dichalcogenide nanosheets. Nat. Chem 5, 263–275, doi:10.1038/nchem.1589 (2013). 23511414

[b7] Gong C. *et al.* Band alignment of two-dimensional transition metal dichalcogenides: Application in tunnel field effect transistors. App. Phys. Lett. 103, doi:10.1063/1.4817409 (2013).

[b8] Yun W. S., Han S., Hong S. C., Kim I. G. & Lee J. Thickness and strain effects on electronic structures of transition metal dichalcogenides: 2H-MX2 semiconductors (M = Mo, W; X = S, Se, Te). Phys. Rev. B 85, 033305, doi:10.1103/PhysRevB.85.033305 (2012).

[b9] Radisavljevic B., Radenovic A., Brivio J., Giacometti V. & Kis A. Single-layer MoS2 transistors. Nat. Nano 6, 147–150, doi:10.1038/nnano.2010.279 (2011). 21278752

[b10] Keyes R. W. The Electrical Properties of Black Phosphorus. Phys. Rev. 92, 580–584, doi:10.1103/PhysRev.92.580 (1953).

[b11] Takahashi T. *et al.* Angle-resolved photoemission study of black phosphorus: Interlayer energy dispersion. Phys. Rev. B 33, 4324–4326 (1986). 10.1103/physrevb.33.4324 9938871

[b12] Vanderborgh C. A. & Schiferl D. Raman studies of black phosphorus from 0.25 to 7.7 GPa at 15 K. Phys. Rev. B 40, 9595–9599, doi:10.1103/PhysRevB.40.9595 (1989). 9991478

[b13] Zhang C. D. *et al.* Surface Structures of Black Phosphorus Investigated with Scanning Tunneling Microscopy. J. Phy. Chem. C 113, 18823–18826, doi:10.1021/jp907062n (2009).

[b14] Cartz L., Srinivasa S. R., Riedner R. J., Jorgensen J. D. & Worlton T. G. Effect of pressure on bonding in black phosphorus. J. Chem. Phys. 71, 1718–1721, doi:10.1063/1.438523 (1979).

[b15] Lei W. *et al.* Oxygen-doped boron nitride nanosheets with excellent performance in hydrogen storage. Nano Energy 6, 219–224, doi:10.1016/j.nanoen.2014.04.004 (2014).

[b16] Jiang J.-W. & Park H. S. Negative poisson’s ratio in single-layer black phosphorus. Nat. Commun. 5, doi:10.1038/ncomms5727 (2014). 25131569

[b17] Low T. *et al.* Plasmons and Screening in Monolayer and Multilayer Black Phosphorus. Phys. Rev. Lett 113, 106802, doi:10.1103/PhysRevLett.113.106802 (2014). 25238376

[b18] Appalakondaiah S., Vaitheeswaran G., Lebègue S., Christensen N. E. & Svane A. Effect of van der Waals interactions on the structural and elastic properties of black phosphorus. Phys. Rev. B 86, 035105, doi:10.1103/PhysRevB.86.035105 (2012).

[b19] Du Y., Ouyang C., Shi S. & Lei M. Ab initio studies on atomic and electronic structures of black phosphorus. J. Appl. Phys. 107, doi:10.1063/1.3386509 (2010).

[b20] Xiao P., Fan X.-L., Liu L.-M. & Lau W.-M. Band gap engineering of FeS2 under biaxial strain: a first principles study. Physical Chemistry Chemical Physics 16, 24466–24472, doi:10.1039/c4cp03453h (2014). 25308322

[b21] Tran V., Soklaski R., Liang Y. & Yang L. Layer-controlled band gap and anisotropic excitons in few-layer black phosphorus. Phys. Rev. B 89, 235319, doi:10.1103/PhysRevB.89.235319 (2014).

[b22] Rudenko A. N. & Katsnelson M. I. Quasiparticle band structure and tight-binding model for single- and bilayer black phosphorus. Phys. Rev. B 89, 201408, doi:10.1103/PhysRevB.89.201408 (2014).

[b23] Buscema M. *et al.* Fast and Broadband Photoresponse of Few-Layer Black Phosphorus Field-Effect Transistors. Nano Letters 14, 3347–3352, doi:10.1021/nl5008085 (2014). 24821381

[b24] Park C. M. & Sohn H. J. Black Phosphorus and its Composite for Lithium Rechargeable Batteries. Adv. Mater. 19, 2465–2468, doi:10.1002/adma.200602592 (2007).

[b25] Sun L.-Q. *et al.* Electrochemical Activity of Black Phosphorus as an Anode Material for Lithium-Ion Batteries. J. Phys. hem. C 116, 14772–14779, doi:10.1021/jp302265n (2012).

[b26] Ma X., Ning G., Qi C., Xu C. & Gao J. Phosphorus and Nitrogen Dual-Doped Few-Layered Porous Graphene: A High-Performance Anode Material for Lithium-Ion Batteries. ACS Appl Mater Interfaces 6, 14415–14422, doi:10.1021/am503692g (2014). 25105538

[b27] Dai J. & Zeng X. C. Bilayer Phosphorene: Effect of Stacking Order on Bandgap and Its Potential Applications in Thin-Film Solar Cells. J. Phys. Chem. Lett 5, 1289–1293, doi:10.1021/jz500409m (2014). 26274486

[b28] Buscema M., Groenendijk D. J., Steele G. A., van der Zant H. S. J. & Castellanos-Gomez A. Photovoltaic effect in few-layer black phosphorus PN junctions defined by local electrostatic gating. Nat. Commun 5, doi:10.1038/ncomms5651 (2014). 25164986

[b29] Fei R. *et al.* Enhanced Thermoelectric Efficiency via Orthogonal Electrical and Thermal Conductances in Phosphorene. Nano. Lett 14, 6393–6399, doi:10.1021/nl502865s (2014). 25254626

[b30] Ma S.-Y., Liu L.-M. & Wang S.-Q. The microstructure, stability, and elastic properties of 14H long-period stacking-ordered phase in Mg-Zn-Y alloys: a first-principles study. Journal of Materials Science 49, 737–748, doi:10.1007/s10853-013-7755-1 (2014).

[b31] Guo H., Lu N., Dai J., Wu X. & Zeng X. C. Phosphorene Nanoribbons, Phosphorus Nanotubes, and van der Waals Multilayers. J. Phys. Chem. C 118, 14051–14059, doi:10.1021/jp505257g (2014).

[b32] Rodin A. S., Carvalho A. & Castro Neto A. H. Strain-Induced Gap Modification in Black Phosphorus. Phys. Rev. Lett 112, 176801, doi:10.1103/PhysRevLett.112.176801 (2014). 24836264

[b33] Fei R. & Yang L. Strain-Engineering the Anisotropic Electrical Conductance of Few-Layer Black Phosphorus. Nano. Lett 14, 2884–2889, doi:10.1021/nl500935z (2014). 24779386

[b34] Gong K., Zhang L., Ji W. & Guo H. Electrical contacts to monolayer black phosphorus: A first-principles investigation. Phys. Rev. B 90, 125441, doi:10.1103/PhysRevB.90.125441 (2014).

[b35] Zhu Z. & Tománek D. Semiconducting Layered Blue Phosphorus: A Computational Study. Phys. Rev. Lett 112, 176802, doi:10.1103/PhysRevLett.112.176802 (2014). 24836265

[b36] Liu Y., Xu F., Zhang Z., Penev E. S. & Yakobson B. I. Two-Dimensional Mono-Elemental Semiconductor with Electronically Inactive Defects: The Case of Phosphorus. Nano. Lett 16, 6782–6786, doi:10.1021/nl5021393 (2014). 25162380

[b37] Kim G. *et al.* Growth of high-crystalline, single-layer hexagonal boron nitride on recyclable platinum foil. Nano. Lett 13, 1834–1839, doi:10.1021/nl400559s (2013). 23527543

[b38] Georgakilas V. *et al.* Functionalization of graphene: covalent and non-covalent approaches, derivatives and applications. Chemical reviews 112, 6156–6214, doi:10.1021/cr3000412 (2012). 23009634

[b39] Goedecker S., Teter M. & Hutter J. Separable dual-space Gaussian pseudopotentials. Phys. Rev. B 54, 1703–1710, doi:10.1103/PhysRevB.54.1703 (1996). 9986014

[b40] Perdew J. P., Burke K. & Ernzerhof M. Generalized Gradient Approximation Made Simple. Phys. Rev. Lett 77, 3865–3868, doi:10.1103/PhysRevLett.77.3865 (1996). 10062328

[b41] Grimme S. Semiempirical GGA-type density functional constructed with a long-range dispersion correction. J. Comput. Chem. 27, 1787–1799, doi:10.1002/jcc.20495 (2006). 16955487

[b42] Monkhorst H. J. & Pack J. D. Special points for Brillouin-zone integrations. Phys. Rev. B 13, 5188–5192, doi:10.1103/PhysRevB.13.5188 (1976).

[b43] Qiao J., Kong X., Hu Z.-X., Yang F. & Ji W. High-mobility transport anisotropy and linear dichroism in few-layer black phosphorus. Nat. Commun 5, doi:10.1038/ncomms5475 (2014). PMC4109013 25042376

